# Invitation for Nominations/Applications for 2021

**DOI:** 10.1093/cdn/nzaa183

**Published:** 2021-01-07

The American Society for Nutrition Foundation (ASNF) awards program honors scientists, clinicians, and scholars for significant achievements in nutrition research and practice. Nominations/applications are now being accepted for the following 2021 Awards will be presented during NUTRITION 2021 LIVE ONLINE from 7–10 June 2021 which will be held virtually.

- Class of 2021 ASN Fellows
- Bray Outstanding Scientific Achievements Award in Obesity Research
- Gilbert A. Leveille Lectureship and Award
- ASN Industry Recognition Award
- Ajinomoto Young Investigator Pilot Grant
- Herbalife Nutrition Scholarship
- ASN Foundation Predoctoral Fellowships for students in nutrition research

Presentation of select awards may be contingent upon funding availability. Awards without current funding are noted; interested partners may contact Keith Dillon [kdillon@nutrition.org; (240) 428–3601] for information.

Due to the cancellation of Nutrition 2020 which was scheduled to be held in Seattle, WA, all senior, mid-career and junior investigator awards that were to be recognized in 2020 will be honored during NUTRITION 2021 LIVE ONLINE. The next call for nomination for these awards will take place during the fall of 2021.

The ASN Foundation's Awards Program is funded through generous individual donors and corporate partners. Additional funds are always needed to help ensure the sustainability of this important program. If you would like to make a charitable donation or sponsor an ASN Foundation award, please visit the ASN website or contact Keith Dillon at kdillon@nutrition.org.

## Nomination/Application Instructions

- Enter the ASN Foundation Portal (https://nutrition.org/asn-foundation/awards/) to submit your nomination(s) and application(s).
- The deadline for receipt of nominations and applications is 22 January 2021.
- Each nomination or application must include (1) a letter of support outlining the basis for the nomination and the significance of the candidate's work, and (2) a brief curriculum vitae for the nominee, including a list of the nominee's publications in refereed journals. Please note that additional requirements for Class of 2021 ASN Fellows can be found at https://nutrition.org/asn-foundation/fellows/.
- All nomination or application materials must be submitted electronically through the ASN Foundation Portal. Incomplete or late submissions will not be considered. For the most up-to-date information regarding the Society awards program, visit https://nutrition.org/asn-foundation/awards/. ASN membership is not required to submit a nomination.
- Nominations and applications for the ASN Industry Recognition Award, Bray Outstanding Scientific Achievements Award in Obesity Research and Class of 2021 ASN Fellows will be retained for 2 years. Nominations and applications received will be considered for 2021 and 2022 awards selection.
- ASN membership is not a requirement for all Society awards but outlining service to the Society is helpful when applicable.
- Any questions, please contact awards@nutrition.org.

## Selection Process

- Confidential award selection juries are appointed each year. Juries rank the candidates based on the award criteria.
- An award is usually given to 1 person, but a jury may recommend that the award be given to 2 or more individuals who collaborated in recent research. Similarly, a jury can determine that there are no deserving candidates in a given year.

## Senior Investigator Lectureships

*Recipients of the following two awards will deliver a lecture on a topic consistent with the award's purpose at NUTRITION 2021 LIVE ONLINE, ASN's Scientific Sessions and Annual Meeting, 7–10 June 2021 which will be held virtually*.

### Bray Outstanding Scientific Achievements Award in Obesity Research

This award will be presented to a physician, clinician or investigator who has made a significant lifetime contribution to the field of obesity research. This award is designed to recognize an individual whose lifetime body of work includes meaningful contributions and/or demonstration of highly original, sustained, and reproducible scholarship that has made major contributions to the understanding of the causes, treatment and/or prevention of obesity. The award is endowed by Dr. George A. Bray and Marilyn M. Bray.

This is the inaugural year for this award.

### The Gilbert A. Leveille Lectureship and Award

This award recognizes outstanding research in nutrition science and food technology. This award was created in 2009, is sponsored by a group of ASN and IFT (Institute of Food Technologists) corporate partners, and is co-administered by ASN and IFT. Nominations for the 2021 award will be accepted by ASN.

Recent recipients include: B Schneeman, M Ferruzzi, RH Liu, GH Johnson.

## Additional Awards

### ASN Industry Recognition Award

This award recognizes significant contributions to the field of nutrition science or practice by an individual from the industry sector (includes commodity boards and industry trade organizations). This award is supported by ASN's Sustaining Partner Roundtable.

This is the inaugural year for this award.

**ASN Fellows**

The ASN Fellows Selection Panel invites applications for the Class of 2021 ASN Fellows. The ASN Fellow designation is the highest honor bestowed by the Society in recognition of significant discoveries and distinguished careers in nutrition. Scientists who have had distinguished careers in the field of nutrition and are at least sixty-five years of age are eligible for consideration.

For more information on the ASN Fellows program, please visit https://nutrition.org/asn-foundation/fellows/.

**Special Awards & Competitions**

ASN offers additional awards for graduate and medical students, faculty, clinicians and researchers. We welcome your applications and sponsorship inquiries. Please visit the ASN website for more information, application deadlines, and sponsorship opportunities.

- Ajinomoto Young Investigator Pilot Grant (application deadline: 22 January 2021, visit https://nutrition.org/asn-foundation/awards/ for more information)
- ASN Foundation Predoctoral Fellowships for students in nutrition research (application deadline: 22 January 2021, visit https://nutrition.org/asn-foundation/awards/ for more information)
- Herbalife Nutrition Scholarship (application deadline: 22 January 2021, visit https://nutrition.org/asn-foundation/awards/ for more information)
- Milton L Sunde Award (for publication in *The Journal of Nutrition* of outstanding experimental, applied, or fundamental research in nutrition that uses an avian species; visit: jn.nutrition.org)
- Science Policy Fellowships

For its annual meeting, ASN awards include the following.

- Clinical Emerging Leader Award Competition
- Emerging Leaders in Nutrition Science Abstract Recognition Awards
- George Bray Obesity Research Student Awards, endowed by George and Marilyn Bray
- Global Nutrition Council Early Career Scholar Award
- Graduate Student Research Award Competition
- Nutrition Translation Award Competition for Early Career Investigators
- Postdoctoral Research Award Competition, endowed by DuPont Nutrition & Health
- Robert Suskind and Leslie Lewinter-Suskind Pediatric Nutrition Student Awards, endowed by Robert Suskind and Leslie Lewinter-Suskind
- Student Interest Group (SIG) Student Awards
- Young Minority Investigator Oral Competition, supported by DSM Nutritional Products

